# The Effect of Preoperative Carbohydrate Loading on Clinical and Biochemical Outcomes after Cardiac Surgery: A Systematic Review and Meta-Analysis of Randomized Trials

**DOI:** 10.3390/nu12103105

**Published:** 2020-10-12

**Authors:** Katarzyna Kotfis, Dominika Jamioł-Milc, Karolina Skonieczna-Żydecka, Marcin Folwarski, Ewa Stachowska

**Affiliations:** 1 Department of Anesthesiology, Intensive Therapy and Acute Intoxications, Pomeranian Medical University, 70-111 Szczecin, Poland; katarzyna.kotfis@pum.edu.pl; 2 Department of Human Nutrition and Metabolomics, Pomeranian Medical University in Szczecin, 71-460 Szczecin, Poland; karzyd@pum.edu.pl (K.S.-Ż.); ewa.stachowska@pum.edu.pl (E.S.); 3 Department of Clinical Nutrition and Dietetics, Medical University of Gdansk, 80-210 Gdansk, Poland; marcinfol@gumed.edu.pl; 4 Home Enteral and Parenteral Nutrition Unit, General Surgery, Nicolaus Copernicus Hospital, 80-210 Gdansk, Poland

**Keywords:** cardiac surgical procedures, coronary artery bypass grafting (CABG), enhanced recovery after surgery (ERAS), carbohydrate loading, insulin, inotropes, fasting

## Abstract

Background and aim: Preoperative fasting leads to metabolic stress and causes insulin resistance in patients undergoing cardiac surgery. The aim of this study was to assess the effect of preoperative oral carbohydrate loading (OCH) on outcome in patients undergoing planned cardiac surgery by systematically reviewing the literature and synthesizing evidence from randomized controlled trials (RCTs). Methods: Systematic search of PubMed/MEDLINE/Embase/Cinahl/Web of Science/ClinicalTrials databases was performed to identify relevant RCTs from databased inception until 05/03/2020. We included studies that compared outcome measures between OCH with control (placebo or standard starvation). We conducted a random-effect meta-analysis of clinical and biochemical parameters. Results: Nine studies (*N* = 9) were included with a total of 507 patients. OCH significantly decreased aortic clamping duration (*n* = 151, standardized mean difference (SMD) = −0.28, 95% confidence interval (CI) = −0.521 to −0.038, *p* = 0.023 and differences in means (DM) = −6.388, 95%CI = −11.246 to −1.529, *p* = 0.010). Patients from treatment groups had shorter intensive care unit (ICU) stay (n = 202, SMD = −0.542, 95%CI = −0.789 to −0.295, *p* < 0.001 and DM = −25.925, 95%CI = −44.568 to −7.283, *p* = 0.006) and required fewer units of insulin postoperatively (n = 85, SMD = −0.349, 95%CI = −0.653 to −0.044, *p* = 0.025 and DM = −4.523, 95%CI = −8.417 to −0.630, *p* = 0.023). The necessity to use inotropic drugs was significantly lower in the OCH group (risk ratio (RR) = 0.795, 95%CI = 0.689 to 0.919, *p* = 0.002). All other primary outcomes did not reveal a significant effect. Conclusions: Preoperative OCH in patients undergoing cardiac surgery demonstrated a 20% reduction in the use of inotropic drugs, a 50% reduction of the length of ICU stay, a 28% decrease in aortic clamping duration and a 35% decrease of postoperative insulin requirement.

## 1. Introduction

### 1.1. Preoperative Fasting

Major cardiac surgery causes metabolic stress and insulin resistance that can be exacerbated by preoperative fasting [1]. Insulin resistance may lead to hyperglycemia and decreased tissue responsiveness to the biological activity of insulin, a metabolic problem that induces catabolic state and may lead to increased morbidity, prolonged hospital and intensive care unit (ICU) stay and decreased survival [2,3,4,5]. Stress hyperglycemia, even in non-diabetic patients, is a marker of stress response in critically ill patients and results from a release of contra-insulin hormones (i.e., glucocorticoids and catecholamines) [6]. Hyperglycemia after cardiac surgery can be detrimental to the heart due to glucose toxicity that causes increased oxidative stress via the hexosamine metabolic pathway and by elevated levels of advanced glycation end-products [7,8,9].

Preoperative fasting, defined as no solid food six hours prior to surgery and no clear liquids two hours prior to surgery, is the standard approach in elective surgery aimed at reducing the risk of aspiration during induction of anesthesia and intubation [10,11]. On the other hand, evidence has shown that fasting not only contributes to catabolic state of stress response related to surgery, but also causes gastrointestinal (GI) problems after surgery and may lead to postoperative delirium and cognitive dysfunction [12,13,14,15,16]. It is noteworthy that monitoring of GI function is very challenging in the ICU [17]. Fasting times are often exceeded due to organizational issues; therefore, limiting the time without oral intake of food or liquids is of major importance in order to improve postoperative outcome and patient satisfaction [18,19,20].

### 1.2. Oral Carbohydrate Loading

One of the measures to improve post-operative outcome is oral carbohydrate loading (OCH) treatment, initiated to optimize the nutritional status of the patient prior to elective surgery as part of the Enhanced Recovery After Surgery (ERAS) pathway [21,22,23]. ERAS is a multimodal, multidisciplinary initiative to improve perioperative care with the effect of substantial improvements in clinical outcomes and cost savings [21]. It is especially relevant to cardiac surgery and includes issues related to human nutrition and metabolism during the preoperative preparation (fasting, preoperative carbohydrate treatment), intraoperative management (blood glucose monitoring and treatment) and postoperative approach (treatment of nausea and vomiting, early nutrition and gastrointestinal stimulation) [22,24,25,26,27].

Many studies have evaluated the effect of preoperative use of an oral drink of simple and/or other digestible carbohydrates on clinical and metabolic outcomes. Their results have shown that OCH decreases postoperative insulin resistance and improves glucose kinetics [28,29], facilitates return of bowel function [13], preserves skeletal muscle mass [30,31], modifies hormonal and metabolic response [32,33,34], prevents surgery-induced immunodepression [35], decreases surgical site infections [36], improves patients’ satisfaction [37] and lowers the total number of complications and the length of hospitalization time [36]. It is important to limit the OCH use for patients without contraindications, including known gastroesophageal reflux disease, disorders of gastric motility or diabetes associated with diabetic gastroparesis [38].

As has been shown by Brady et al., clear fluids are removed from the stomach within one hour (up to 90 min) [39]. Administration of clear liquids up to two hours before elective operations does not expose patients to greater risk of aspiration, regurgitation or related morbidity as compared to those fasted for 12 h or longer before surgery [39]. For this reason, anesthesiology societies issued recommendations that allow patients to drink an oral carbohydrate (OCH) drink. The procedure called “oral carbohydrate loading” typically allows the patient to drink a volume of 400–800 mL of OCH the night before surgery and a volume of 200–400 mL up to 2 h before elective surgery, with the exception of patients with special risk of aspiration, e.g., with gastro-esophageal reflux or delayed gastric emptying (for any reason) [39,40]. Also fruit-based drinks (i.e., 400 mL of over-the-counter fruit-based lemonade, containing 48 g carbohydrates, mainly fruit-associated saccharides, 805 mOsm/kg) or drinks enriched with glutamine (50 g maltodextrin plus 40 g GLN), antioxidants, and green tea extract (70 g), Vitamin C (750 mg), Vitamin E (250 mg), Selenium (150 mg), Zinc (10 mg), β-Carotene (5 mg), Green tea extract (1 g), Glutamine (15 g) and Maltodextrin/saccharose (50 g) may be considered as a safe alternative to CHO drink with no difference in the gastric emptying time [41,42,43]. Typical preoperative oral carbohydrate-rich drink (e.g., for colorectal patients) is a hypo-osmolar solution and contains 12.5 g·100 mL^−1^ carbohydrate (12% monosaccharide, 12% disaccharide, 76% polysaccharide, 285 mOsm kg^−1^) [44] or 12.5 g·100 mL^−1^ carbohydrate (2.1 g·100 mL^−1^ monosaccharide, 10 g·mL^−1^ polysaccharide, 240 mOsm, sodium 50 mg·mL^−1^, potassium 122 mg·mL^−1^, chloride 6 mg·mL^−1^, calcium 6 mg·mL^−1^, phosphorus and magnesium 1 mg·mL^−1^, pH of 4.9) [45].

Many studies regarding OCH have been performed involving a wide range of outcomes, but their quality is unsatisfactory, therefore the evidence that preoperative carbohydrate treatment reduces major endpoints—hospital stay and mortality—is still lacking. Despite the high importance of the problem, a meta-analysis of studies concentrating on the effects of preoperative carbohydrate treatment on clinical and metabolic endpoints in cardiac surgery has not been performed.

### 1.3. Specific Aims

The aims of this meta-analysis were to evaluate the influence of pre-operative carbohydrate loading in patients undergoing elective cardiac surgery on clinical (length of hospital and ICU stay, mortality, post-operative surgical complications, post-operative non-surgical complications, post-operative nausea and vomiting (PONV)) and metabolic outcomes (i.e., glycemia, development of postoperative insulin resistance, effects of starvation).

## 2. Materials and Methods

We performed a systematic review and a meta-analysis of randomized controlled trials (RCTs) in accordance with the Preferred Reporting Items for Systematic Reviews and Meta-Analyses (PRISMA) guidelines [46].

### 2.1. Search Strategy and Inclusion Criteria

At least two independent authors (K.S.-Ż., D.J.-M., M.F.) searched PubMed/MEDLINE/Embase/Cinahl/Web of Science/ClinicalTrials from database inception until 05/03/2020 without language restriction for randomized controlled trials (RCTs) comparing carbohydrate loading with placebo/fasting to counteract particular clinical and biochemical outcomes in patients undergoing cardiac surgery.

The following search terms were used in PubMed: (surgery* OR operation* OR operation care OR operative intervention OR operative surgical procedure OR operative treatment OR research surgery OR resection OR surgery, operative OR surgical care OR surgical correction OR surgical exposure OR surgical intervention OR surgical management OR surgical operation OR surgical practice OR surgical procedures, operative OR surgical repair OR surgical research OR surgical restoration OR surgical service OR surgical therapy OR surgical treatment) AND (carbohydrate load* OR oral carbohydrates OR “carbohydrate solution” OR “carbohydrate drinks”) AND (RCT OR random*) AND “humans”.

In Embase, the following search string was used: (‘surgery’/exp OR ‘operation’ OR ‘operation care’ OR ‘operative intervention’ OR ‘operative surgical procedure’ OR ‘operative treatment’ OR ‘research surgery’ OR ‘resection’ OR ‘surgery’ OR ‘surgery, operative’ OR ‘surgical care’ OR ‘surgical correction’ OR ‘surgical exposure’ OR ‘surgical intervention’ OR ‘surgical management’ OR ‘surgical operation’ OR ‘surgical practice’ OR ‘surgical procedures, operative’ OR ‘surgical repair’ OR ‘surgical research’ OR ‘surgical restoration’ OR ‘surgical service’ OR ‘surgical therapy’ OR ‘surgical treatment’) AND (‘carbohydrate loading’ OR ‘oral carbohydrates’ OR ‘carbohydrate solution’) AND (‘randomized controlled trial’/exp OR ‘controlled trial, randomized’ OR ‘randomized controlled study’ OR ‘randomized controlled trial’ OR ‘randomized controlled study’ OR ‘randomized controlled trial’ OR ‘trial, randomized controlled’) AND “humans”.

For the ClinTrials.Gov search, the following two search terms were utilized: carbohydrate loading AND surgery. The electronic search was supported by a manual review of the reference lists from relevant reviews and publications.

The following inclusion criteria were applied:

(i) Randomized controlled trial,
(ii) Populations containing >15 patients,
(iii) Intervention comprising of oral carbohydrate loading maximum 2 h prior to surgery,
(iv) Randomization to carbohydrate loading versus fasting/placebo (e.g., water),
(v) Available meta-analyzable change score/endpoint data on any biochemical and clinical outcomes, in particular any of the following: surgical stress response (e.g., white blood cells count, C-reactive protein (CRP) concentration, proinflammatory cytokines levels), postoperative complications (e.g., postoperative nausea and vomiting, surgical site infection, GI tract complications, blood loss), non-surgical complications (e.g., pneumonia/urinary tract infections, delirium, antibiotic therapy duration, insulin treatment), mortality, ICU length of stay, inotropic drugs usage, time to extubation, atrial fibrillation and arrythmia.

We excluded studies that randomized patients suffering from insulin-dependent diabetes (Type 1 diabetes mellitus—T1DM). Data from more than 2 arm studies (studies with more than one type of intervention) were abstracted separately for comparators [47].

### 2.2. Data Extraction

Data on sponsorship, blinding, setting, focus of the study, as well as patient and intervention characteristics were independently extracted in accordance with the Preferred Reporting Items for Systematic Reviews and Meta-Analyses (PRISMA) [46] standard by two independent investigators (D.J.-M., K.S.-Ż.). For evaluation of the risk of bias (ROB) [48], we assumed that the higher the number of low risk-of-bias assessments, the greater the quality of the study. Data from figures was extracted by means of WebPlotDigitizer software (https://automeris.io/WebPlotDigitizer/). Whenever data were missing for the review, authors were contacted for additional information twice, via corresponding authors’ emails, two weeks apart. Inconsistencies were resolved by the first author (K.K.), who acted as a clinical guarantor of the article.

### 2.3. Outcomes

Primary outcomes were: acute atrial fibrillation (AAF), aortic clamping time (AC), acute myocardial infarction (AMI), any infectious complications, arrythmia, blood loss, cardio-pulmonary bypass (CPB) duration, duration of mechanical ventilation, duration of surgery, exogenous insulin treatment, intensive care unit (ICU) stay, inotropic drugs usage, postoperative nausea and vomiting (PONV), vasoactive drugs usage and transfusion requirement.

Secondary outcomes were: bronchial aspiration during induction of anesthesia, stroke, in-hospital mortality, thirst, any complication intra-operatively, post-surgery complication, reoperation, encephalic vascular accident, hospital length of stay, intra-operative and post-operative gastric drainage, Homeostasis Model Assessment for Insulin Resistance (HOMA-IR) index endpoint, CRP endpoint, interleukin-6 (IL-6) endpoint and blood glucose level post-operatively.

### 2.4. Data Synthesis and Statistical Analysis

We conducted a random effects [49] meta-analysis of outcomes for which ≥3 studies contributed data, using Comprehensive Meta-Analysis V3 (http://www.meta-analysis.com). We evaluated study heterogeneity using the chi-square test of homogeneity, with *p* < 0.05 indicating significant heterogeneity. All analyses were two-tailed with alpha = 0.05. Group differences in continuous outcomes were analyzed as the pooled standardized mean difference (SMD) or for significant effects and where relevant, as differences in means (DM) in endpoints using observed-cases (OC) data. Categorical outcomes were analyzed by calculating the pooled risk ratio (RR). At last, funnel plots and Egger’s regression test [50] followed by the Duval and Tweedie’s trim and fill method [51] to quantify whether publication bias could have influenced the results were done.

### 2.5. Risk of Bias

The quality of each study methodology was classified by two independent investigators (E.S. and D.J.-M.) based on selection, performance, attrition and reporting bias assessment. This was done by means of the Cochrane Collaboration’s tool [48]. We arbitrarily assumed that the quality of a study increased along with the higher number of low risks of bias assessments.

### 2.6. GRADE (Grading of Recommendations, Assessment, Development and Evaluation)—Quality of Evidence Assessment

The GRADE approach enables the assessment of quality of evidence in systematic reviews and guidelines. Overall quality of the evidence for each outcome was graded as high, moderate, low or very low, depending on the assessment of the following domains: risk of bias, inconsistency, indirectness, imprecision and publication bias [52,53]. The GRADEpro software was used (https://gdt.gradepro.org/app/).

## 3. Results

### 3.1. Search Results

**Figure 1:**
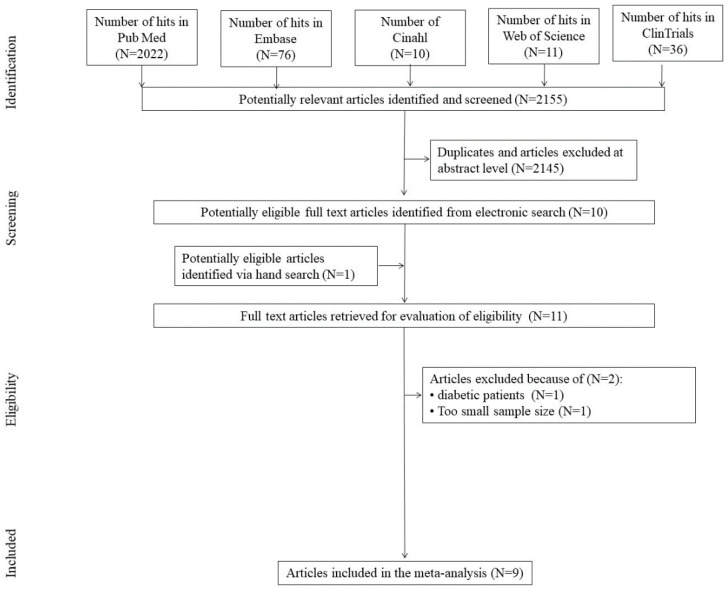
Preferred Reporting Items for Systematic Reviews and Meta-Analyses (PRISMA) study flowchart depicting search strategy and study selection.

The initial search yielded 2155 hits. A total of 2145 studies were excluded, being duplicates and/or after evaluation on the title/abstract level. There was 1 additional article identified via hand search. Overall, 11 full-text articles were incorporated into the final, abstraction level. Of those, 2 were excluded due to not fitting inclusion criteria: the reasons for exclusion were diabetes (*N* = 1) and too small sample size (*N* = 1) (Figure 1). This yielded 9 studies that were included in the meta-analysis. However, in two trials [54,55], the same study cohort was reported with a percent of identical results, thus in present work, these results were not used in duplicate where applicable.

### 3.2. Study, Patient and Treatment Characteristics

Altogether, nine studies (*N* = 9) were included [45,47,54,55,56,57,58,59,60] comprising 11 interventions. However, as mentioned previously, two studies comprised the same study group and presented similar results in separate papers, thus the numbers of studies and interventions are 8 and 10, respectively. These were double-blind [54,55,56] or open-label prospective trials [45,47,57,58,59]. In the majority of studies [45,47,54,55,56,57,58], 12.5% oral carbohydrate (OCH) solution was used. In two studies, 12.8% [59] and 12.6% [60] OCH was ingested. Patients received a different amount of OCH drink, varying from 200 to 1200 mL, either once before the surgery or at various times. The most frequent comparator in the studies included into the qualitative synthesis was fasting [45,47,57,58,59,60]. In three trials (including a duplicate study by Feguri et al. 2017 and Feguri et al. 2019), patients received water instead of OCH load [54,55,56].

A total of 507 patients took part in the included trials. A mean number of randomized patients was equal to 63 per trial, while the analyzed group comprised of 62 patients on average. These were predominantly men (*n* = 469, 76.3%), aged between 57 and 68.5 years. In the majority of studies, coronary artery bypass grafting with cardio-pulmonary bypass and general anesthesia with endotracheal intubation was performed [45,47,54,55,56,57,58]. A subgroup of patients in a study by Sokolic et al. [60] underwent off-pump coronary artery bypass grafting (OPCAB). In a study by Lee et al. [59], OPCAB revascularization was also performed. The mean number of low risks of bias assessments was 4.5, with the highest quality scored at 6 and lowest scored at 3. Only one study scored high risk for allocation concealment (selection bias) and blinding of participants and personnel (performance bias) [57]. The highest number of domains (four) assessed as unclear risk of bias was obtained by four studies [45,47,58,60]. The risk of bias assessments for each study (listed in Table 1) is presented in Supplemental Table S1 Study and patients’ characteristics are shown in Table 1.

### 3.3. OCH Effects on Clinical and Biochemical Parameters

There were no trials retrieved from the search that provided data for all of the primary outcomes. Primary outcomes that were subjected to meta-analysis included: aortic clamping time, arrythmia, cardio-pulmonary bypass (CPB) duration, ICU length of stay, blood loss, duration of mechanical ventilation, duration of surgery, exogenous insulin treatment in the ICU postoperatively, arrythmia, acute myocardial infarction (AMI), acute atrial fibrillation (AAF), any infectious complications, inotropic drugs usage, postoperative nausea and vomiting (PONV), transfusion requirement and vasoactive drugs usage. All secondary outcomes were not meta-analyzable.

**Figure 2:**
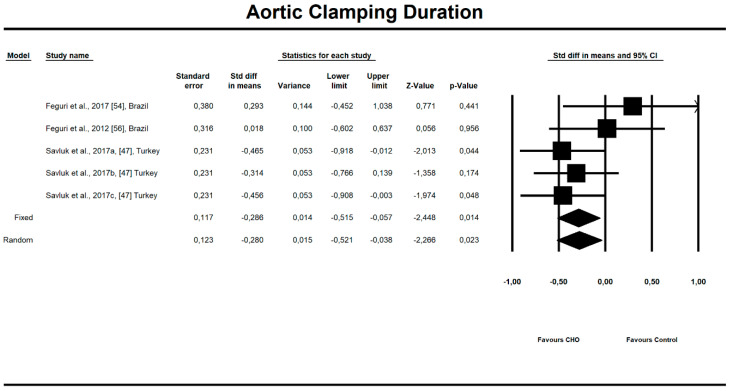
An effect size standardized mean difference, for aortic clamping (AC) duration (minutes) in patients taking oral carbohydrates (OCH) drinks vs. controls. Std diff in means—standardized mean difference; 95%Cl—95% confidence interval.

**Figure 3:**
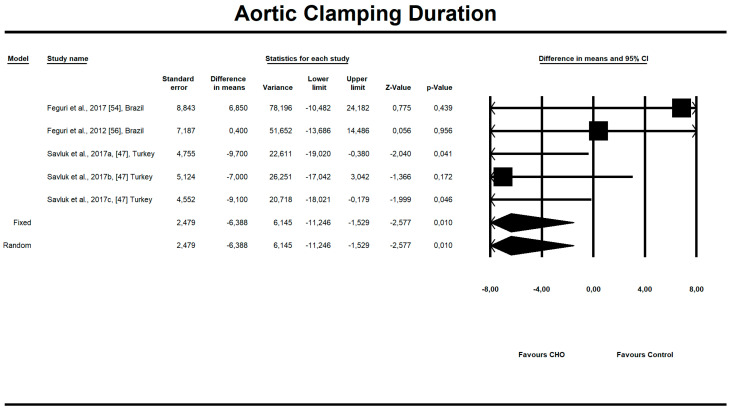
An effect size, difference in means, for aortic clamping (AC) duration (minutes) in patients taking oral carbohydrates (OCH) drinks vs. controls. 95%Cl—95% confidence interval.

**Figure 4:**
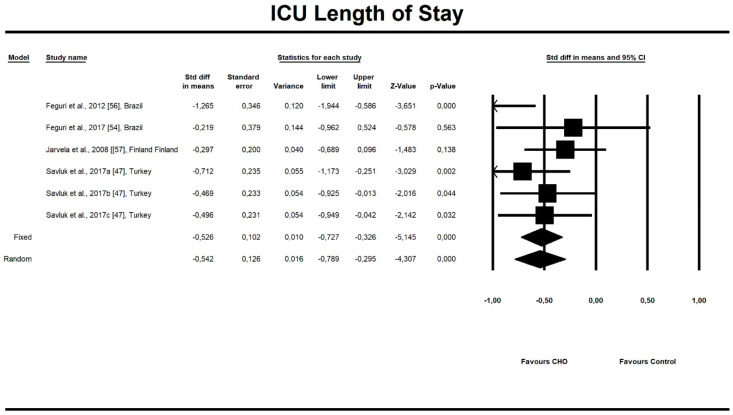
An effect size standardized mean difference, for intensive unit care (ICU) stay (hours) in patients taking oral carbohydrates (OCH) drinks vs. controls. Std diff in means—standardized mean difference; 95%Cl—95% confidence interval.

**Figure 5:**
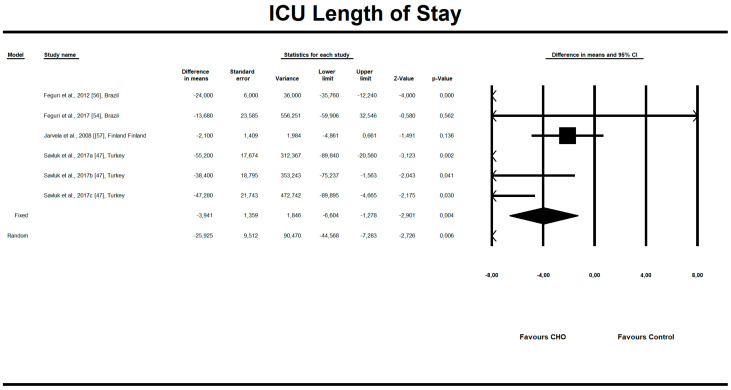
An effect size, difference in means, for intensive care unit (ICU) stay (hours) in patients taking oral carbohydrates (OCH) drinks vs. controls. 95%Cl—95% confidence interval.

**Figure 6:**
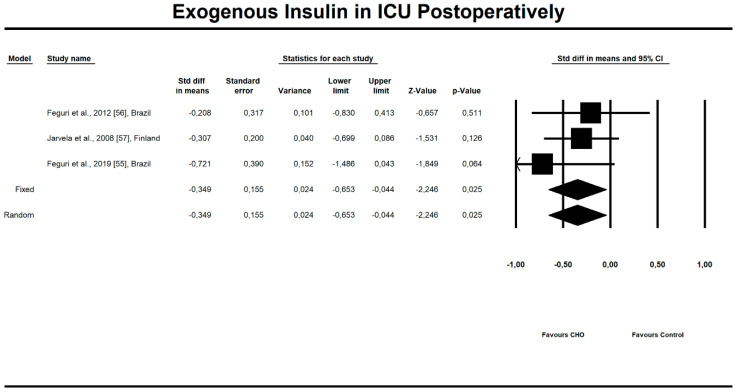
An effect size standardized mean difference, for exogenous insulin units (IU) in intensive care unit (ICU) postoperatively in patients taking oral carbohydrates (OCH) drinks vs. controls. Std diff in means—standardized mean difference; 95%Cl—95% confidence interval.

**Figure 7:**
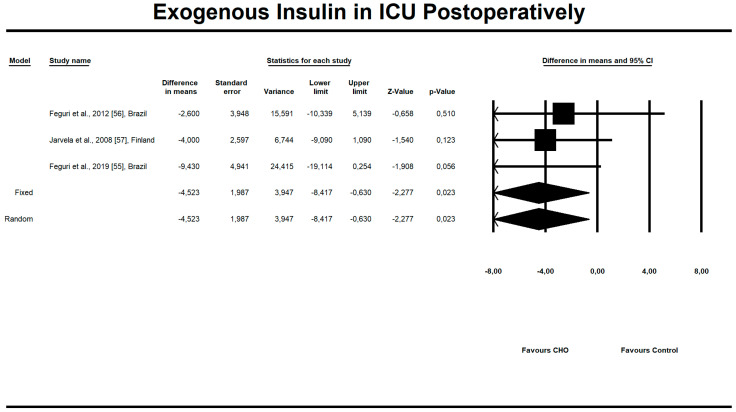
An effect size, difference in means, for exogenous insulin units (IU) in intensive care unit (ICU) postoperatively in patients taking oral carbohydrates (OCH) drinks vs. controls. 95%Cl—95% confidence interval.

**Figure 8:**
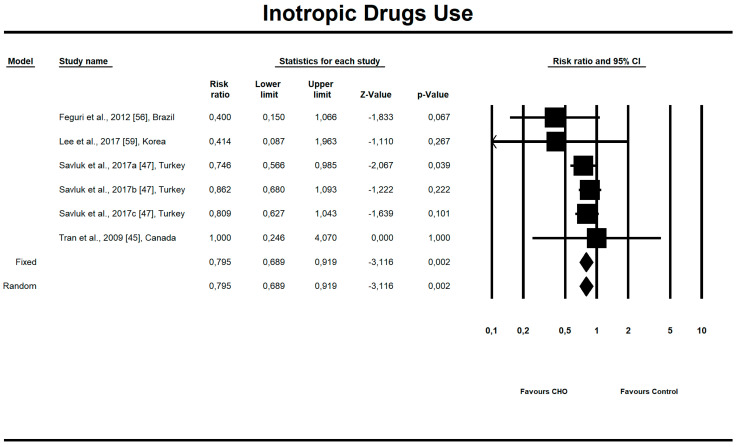
An effect size, risk ratio, for inotropic drugs use in patients taking oral carbohydrates (OCH) drinks vs. controls.

Out of all evaluated indices, the OCH intake significantly affected the duration of aortic clamping, postoperative insulin dose in the ICU, along with the length of ICU stay and finally, the use of inotropic drugs. The OCH drink significantly decreased the aortic clamping (AC) duration (minutes) (*n* = 151, SMD = −0.28, 95% confidence interval (CI) = −0.521 to −0.038, *p* = 0.023 and differences in means (DM) = −6.388, 95%CI = −11.246 to –1.529, *p* = 0.010). Patients from treatment groups had shorter length of ICU stay (hours) (*n* = 202, SMD = −0.542, 95%CI = −0.789 to −0.295, *p* < 0,001 and DM = −25.925, 95%CI = −44.568 to −7.283, *p* = 0.006) and required fewer units of insulin postoperatively (IU) (*n* = 85, SMD = −0.349, 95%CI = −0.653 to −0.044, *p* = 0.025 and DM = −4.523, 95%CI = −8.417 to −0.630, *p* = 0.023). Similarly, the necessity to use inotropic drugs was significantly lower in OCH-treated patients as compared with controls (risk ratio (RR) = 0.795, 95%CI = 0.689 to 0.919, *p* = 0.002). For these results, the forest plots are presented in Figure 2, Figure 3, Figure 4, Figure 5, Figure 6, Figure 7 and Figure 8. In one case, the Egger’s test did indicate a significant publication bias (*p* = 0.007). After applying the Trim and Fill method, the statistics remained unchanged (missing studies to right of mean). All other primary outcomes did not reveal a significant effect.

The Supplementary Tables S2 and S3 present all other meta-analyzed results. Other not meta-analyzable outcomes abstracted by the authors are depicted in Table 2 and Table 3.

### 3.4. The Quality of Evidence Assessment (GRADE)

All studies in the meta-analysis were RCTs; therefore, the outcomes start as high-quality evidence. Three out of four outcomes’ evidence are moderate quality (AC duration, units of the exogenous insulin and the ICU stay) and were downgraded because of the overall low quality of the studies (ROB), detected publication bias, inconsistency or imprecision. The evidence of use of inotropic drugs was graded as low. The GRADE assessment of evidence of statistically significant outcomes: AC duration, length of ICU stay, units of exogenous insulin postoperatively and inotropic drugs, is presented in Table 4. The quality of evidence assessment of all other results are provided in Supplementary Table S4.

## 4. Discussion

### 4.1. Principal Findings

To our knowledge, this study is the first systematic review and meta-analysis of RCTs investigating the effect of oral carbohydrate treatment on outcome in patients undergoing cardiac surgery. This meta-analysis included 9 moderate randomized controlled trials, comprising 11 interventions, with a total number of 507 patients. The results show that oral preoperative carbohydrate treatment in patients undergoing elective cardiac surgery demonstrated a significant 20% reduction in the use of inotropic drugs, nearly 50% reduction of the length of ICU stay, a 28% decrease in the aortic clamping duration time and a 35% decrease of the postoperative insulin requirement in the cardiac ICU. The interventions differed between studies, yet preoperative OCH was safe (no occurrence of drink-related complications), associated with reduced development of postoperative insulin resistance, but the latter was not associated with any effect on surgical complications.

### 4.2. Results in the Context of other Meta-Analyses

As mentioned previously, no other meta-analysis concentrated solely on the cardiac surgery population. The results of analyses that included all different types of surgical procedures are inconsistent. An analysis performed by Amer et al. showed that OCH loading before elective surgery conferred a small reduction in the length of postoperative hospital stay compared with fasting, and no benefit in comparison with water or placebo [61]. A meta-analysis provided by Li et al. in 2012 reported data provided by 22 studies, with only two regarding elective cardiac surgery [36]. According to their analysis, the OCH increased the insulin and glucose levels on the first postoperative day as compared to patients who fasted overnight. The pooled results showed a greater decline in the level of insulin at anesthesia induction and a smaller increase in the level of glucose at the end of surgery. The results also showed fewer episodes of a decrease of the insulin sensitivity index in the postoperative period in the OCH loading group as compared with the group with placebo. The quality of most of the trials was poor. No aspiration was reported by the authors. The two RCTs included by Li et al. [36] that reported data regarding cardiac surgery (Jarvela et al. [57] and Breuer et al. [62]) and comparing OCH with overnight fasting or placebo showed no significant differences in the levels of glucose at the anesthesia induction, the length of ICU and hospital stay or the incidence of PONV. In cardiac surgery patients, there were no significant differences in the rate of preoperative hunger, nausea, or mouth dryness in the OCH group as compared with placebo or overnight fasting, yet less thirst was reported in the carbohydrate drink group as compared with overnight fasting [36].

One other analysis yielded results similar to our report. A meta-analysis performed in the year 2012 by Awad et al. [63] of twenty-one randomized controlled trials (1685 patients) regarding patients undergoing all types of elective surgery included only two studies involving cardiac surgery patients—Rapp-Kesek et al. [58] and Järvelä et al. [57]. No statistically significant differences in the length of hospitalization (mean difference (MD) −0.19, 95%CI −0.46 to 0.08; 12 trials; Ι^2^ = 83%) or the incidence of postoperative complications (RR 0.88, 95%CI 0.50 to 1.53; nine trials; Ι^2^ = 41%) was reported. A statistically significant reduction in the length of hospital stay was found among patients who received CHO prior to open abdominal surgery as compared with the controls (MD −1.08, 95%CI −1.87 to −0.29; seven trials; Ι^2^ = 60%). An increase in the length of hospital stay was reported among patients who received OCH treatment prior to orthopedic surgery, as compared with the controls (MD 0.48, 95%CI 0.23 to 0.73; two trials; Ι^2^ = 0%). Six out of seven clinical trials that evaluated postoperative insulin resistance reported significantly lower rates among the treatment groups. There was no evidence of publication bias and no occurrences of drink-related pulmonary complications. The authors concluded that the preoperative carbohydrate treatment may be linked to a reduction in the length of hospital stay in patients undergoing major abdominal surgery, attenuation of insulin resistance in all patients, however, the strength of the evidence was unsatisfactory (low for primary meta-analysis and moderate for subgroup analyses) [63].

### 4.3. Strengths of the Meta-Analysis

This is the first meta-analysis to examine the effects of preoperative carbohydrate treatment on clinical outcomes in patients undergoing elective coronary artery bypass grafting surgery. Therefore, for the primary outcomes of acute atrial fibrillation (AAF), aortic clamping time (AC), acute myocardial infarction (AMI), any infectious complications, arrythmia, blood loss, cardio-pulmonary bypass (CPB) duration, duration of mechanical ventilation, duration of surgery, exogenous insulin treatment, intensive care unit (ICU) stay, inotropic drugs usage, postoperative nausea and vomiting (PONV), vasoactive drugs usage and transfusion requirement, the data from this group of patients is of clinical importance and comprises the largest population of patients available in the literature. The assessment of the quality of individual studies and the reported outcomes further strengthens the methodology.

### 4.4. Limitations of the Meta-Analysis

This meta-analysis has some limitations. The main limitation of this meta-analysis is the quality of included studies graded as moderate. The patient populations were small and significant heterogeneity in the study design occurred, such as inclusion and exclusion criteria, timing of randomization and the type and timing of the intervention. Moreover, the definitions of outcomes and postoperative complications vary between studies. Similarly, different measures in the included studies were used to define insulin resistance, making the comparisons difficult. Not all outcomes were reported by the included studies, we included studies that reported at least one outcome of interest. These limitations resulted in a small number of studies eligible for inclusion in this meta-analysis.

### 4.5. Implication for Clinical Practice

The idea of preoperative carbohydrate loading is a part of multimodal protocols gathering guidelines for perioperative management of surgical patients. Most data concerning cardiac surgery were extrapolated from studies on patients undergoing abdominal surgery. Starting from Kehlet’s studies in 2002, several fast-track surgery protocols have been proposed like the ACERTO Project (Acceleration of Total Postoperative Recovery) or ERAS [64,65,66]. Studies confirm that compliance with only a few elements of those protocols results in fewer benefits for outcome improvement. CHO loading, early nutrition support and avoiding fluid overload are key factors contributing to the decreased length of hospital stay, number of postoperative complications and readmission rates in abdominal surgery [67,68,69].

Postoperative nausea and vomiting (PONV) are defined as either occurring within the first 24 to 48 h after surgery. A systematic review and meta-analysis provided by Amirshahi et al. showed that the prevalence of nausea, vomiting and combined PONV among surgical patients was 7%, 4% and 8%, respectively [70]. Inconsistent data are showing that PONV is related to the type of surgery, with a high occurrence in breast and abdominal surgery. Several scales to predict postoperative PONV are proposed in the studies summarizing known risk factors [71,72,73]. Three studies included in the meta-analysis reported no influence of OCH loading on PONV incidence. However, no data of PONV risk factors like history of migraine, motion sickness or history of PONV were recorded. Perioperative use of drugs preventing PONV was also not described in study protocols.

The heterogeneity of the OCH loading intervention was high. The following OCH administration protocols were proposed: (a) 8 h fasting for solid foods, then 2 h fasting for liquids plus 200 mL of oral intake of 12.5% maltodextrin dissolved in water [54,55,60], (b) fasting overnight, then at 6:00 h, 2 h before induction of anesthesia, the patients ingested 400 mL of fluid (12.5% carbohydrates, 50 kcal/100 mL, 240 mOsm/L [57]). (c) On the evening prior to surgery (between 9:00 and 11:00 p.m.), 400 mL dose of iso-osmolar preoperative fluid containing 12.8% CHO (0.5 kcal mL^−1^, 290 mOsm kg^−1^) and an additional 400 mL CHO within 2 to 3 h prior to surgery [58,59], (d) 8 h before the procedure, 800 mL of a carbohydrate-rich drink containing 12.5 g/100 mL of carbohydrates (12% monosaccharides, 12% disaccharides, 76% polysaccharides and 285 mOsm/kg^−1^), and 2 h before the surgery, 400 mL of carbohydrates or 400 mL of the same CHO drink (containing 12.5 g/100 mL carbohydrates) 8 h before the procedure only, or, 400 mL of carbohydrates 2 h before the surgery only [47], (e) 800 mL of an iso-osmolar preoperative supplement (12.5 g/100 mL CHO) between 9:00 and 11:00 p.m. the evening before surgery and 400 mL 2 h before the procedure [45] and (f) fasting for solids starting at 22 h the day before surgery and then 400 mL 6 h and 200 mL 2 h before induction of anesthesia [56].

Despite the recommendations from ERAS Guidelines, not all studies included in our analysis reported preoperative levels of Hba1c. Halkos et al. [74] reported that a Hba1c level of 8.6% was associated with 4-fold greater mortality in elective coronary artery bypass grafting. Approximately 10% of patients qualified for cardiac surgery may have undiagnosed diabetes [64,74,75], therefore ERAS guidelines recommend the assessment of HbA1c in the preoperative period. Only two studies included in the meta-analysis reported baseline Hba1c levels. Uncontrolled glucose metabolism may influence the effect of CHO loading. Jarvela et al. reported that patients with a HbA1c value of 6% or more had higher glucose levels and needed more insulin compared with the patients with HbA1C < 6% [57].

Postoperative introduction of oral, enteral or parenteral nutrition was not mentioned in the studies included in our meta-analysis. Rapp-Kesek [58] reported providing breakfast on the first postoperative day, and Jarvela et al. [57] reported removal of the naso-gastric tube together with extubation. Studies show that preoperative nutritional status is an independent risk factor of postoperative complications and mortality in cardiac surgery [76,77].

### 4.6. Implications for Future Research

As the quality of evidence presented in this meta-analysis is moderate, it is obvious that further well-designed randomized trials including homogenous groups of patients are necessary to specifically examine the biochemical and clinical effects of preoperative carbohydrate loading in cardiac surgery. Further studies should be performed in patients with sufficiently long postoperative length of stay in the ICU and in the hospital. It must be noted, however, that further randomized clinical trials regarding preoperative carbohydrate loading as the only intervention may prove difficult to perform as this intervention is not used in isolation. In majority of cases, OCH treatment has been incorporated into the ERAS protocols, therefore the effect associated with preoperative carbohydrate loading demonstrated in these studies may be difficult to distinguish from the effect of other interventions within ERAS.

## 5. Conclusions

Based on the findings of this meta-analysis, preoperative oral carbohydrate treatment in patients undergoing elective cardiac surgery demonstrated a significant 20% reduction in the use of inotropic drugs, nearly 50% reduction of the length of ICU stay, a 28% decrease in the aortic clamping duration and a 35% decrease of the postoperative insulin requirement in the cardiac ICU. However, because the quality of evidence was moderate, further well-designed randomized trials are warranted. Moreover, the results of this meta-analysis must be interpreted with caution due to heterogeneity of the included studies. Most importantly, preoperative OCH loading is a safe, simple and cheap intervention, associated with no or minimal harm, and therefore, the use of OCH should be recommended in cardiac surgery, with careful consideration for contraindications, such as diabetes or gastrointestinal reflux disease.

## Figures and Tables

**Table 1:** Study characteristics.

No.	Overall Study Characteristics (First Author, Year, Country)	Study Design			Intervention			Patients Characteristics		
		Blinding/ROB	Focus of the Study	Surgery Technique/Anesthesia	OCH Specification, %	Oral Dose, mL	Comparator	R/A (n)	Age—Years; Mean (SD); Median	Male, %/T2DM, %
1	Feguri et al., 2017, Brazil [54]	DB/6	morbidity (especially POAF) in ICU patients	CABG with CPB/GA with ETT	12.5	200 *	water	30/28	62.1 (9.7)	78.6/25
2	Feguri et al., 2019, Brazil [55]	DB/6	morbidity, blood glucose, inflammation, recovery	CABG with CPB/GA with ETT	12.5	200 *	water		62.1 (9.7)	78.6/25
3	Feguri et al., 2012, Brazil [56]	DB/5	perioperative glycemic control and IR of nondiabetic patients	CABG with CPB/GA with ETT	12.5	600 ^†^	water	40/40	58.5 (7.2)	65/0
4	Jarvela et al., 2008, Finland [57]	OL/4	perioperative insulin requirements in non-diabetic patients	CABG with CPB/GA with ETT	12.5	400 *	fasting	101/101	65.4 (10.2)	83.2/0
5	Lee et al., 2017, Korea [59]	OL/6	insulin resistance and free-fatty acid (FFA) concentrations	OPCAB/GA with ETT	12.8	800 ^‡^	fasting	60/57	64.5 (8.5)	86/0
6	Savluk et al., 2017a, Turkey [47]	OL/3	postoperative insulin requirements, postoperative patient discomfort, inotropic support, length of the ICU stay, the duration of postoperative mechanical ventilation	CABG with CPB/GA with ETT	12.5	1200 ^§^	fasting	77/77	58 (11.6)	83/0
	Savluk et al., 2017b, Turkey [47]			CABG with CPB/GA with ETT	12.5	400 ^¶^	fasting	76/76	57.5 (11.5)	81.2/0
	Savluk et al., 2017c, Turkey [47]			CABG with CPB/GA with ETT	12.5	400 *	fasting	77/77	57 (11)	81.8/0
7	Sokolic et al., 2019a, Croatia [60]	OL/3	frequency and perforin expression in peripheral blood lymphocytes	OPCAB/GA with ETT	12.6	200*	fasting	40/40	66 ^‡‡^; 67.5 ^§§^	72.5/0
	Sokolic et al., 2019b, Croatia [60]			CABG with CPB/GA with ETT	12.6		fasting	40/40	68.5 ^‡‡^; 66 ^§§^	67.5/0
8	Tran et al., 2013, Canada [45]	OL/3	glucose and insulin levels, insulin resistance, markers of inflammation (CRP, IL-6), FFA levels, time of mechanical ventilation, incidence of infection, blood transfusions, LOS in the ICU, LOS in the hospital, subjective feelings of discomfort (VAS)	CABG with CPB/GA with ETT	12.5	1200 **	fasting	26/26	55 ^‡‡^; 59 ^§§^	80.8/0
9	Rapp-Kesek et al., 2007, Sweden [58]	OL/3	Muscle strength, insulin resistance, stress hormone response	CABG with CPB/GA with ETT	12.5	800 ^††^	fasting	18/18	72 (1.4)	nd/0

* 2 h before the surgery, ^†^ 400 mL—6 h before the surgery, ^‡^ 400 mL—between 9:00 and 11:00 p.m. in the evening before the surgery, and 400 mL—2 h to 3 h before the surgery, and 200 mL 2 h before induction of anesthesia, ^§^ 800 mL—8 h before the surgery and 400 mL—2 h before the surgery, ^¶^ 8 h before the surgery, ** 800 mL—at the evening before surgery between 21:00 and 23:00 h, and 400 mL consumed over a ten minute period—2 h before the surgery, ^††^ 400 mL in the evening before surgery and 400 mL in the morning on the day of surgery, ^‡‡^ treatment group, ^§§^ control group A—number of analyzed patients. CABG with CPB—Coronary Artery Bypass Grafting with Cardio-Pulmonary Bypass, OCH—oral carbohydrate, CRP—C-reactive protein, DB—double blinding, FFA—free-fatty acid, GA with ETT—General Anesthesia with Endotracheal Intubation, ICU—intensive care unit, Il-6—interleukin 6, LOS—length of stay, nd—no data, OL – open label study, OPCAB—Off-Pump Coronary Artery Bypass Grafting, POAF—postoperative atrial fibrillation, R—number of randomized patients, ROB—risk of bias, as number of Low assessments, SD—standard deviation, SB—single blinding, T2DM—type 2 diabetes mellitus, VAS—visual analog scale.

**Table 2:** Qualitative clinical outcomes that were not subjected to meta-analysis. Values are number of patients.

Outcomes	Treatment Group		Control Group		References
	Cases	*n*	Cases	*n*	
Bronchial aspiration during induction of anesthesia	0	14	0	14	Feguri et al., 2017, Brazil [54]
	0	14	0	14	Feguri et al., 2019, Brazil [55]
	0	20	0	20	Feguri et al., 2012, Brazil [56]
	0	50	0	51	Jarvela et al., 2008, Finland [57]
Stroke	0	14	1	14	Feguri et al., 2017, Brazil [54]
	0	20	0	20	Feguri et al., 2012, Brazil [56]
In-hospital mortality	0	14	1	14	Feguri et al., 2017, Brazil [54]
	0	20	0	20	Feguri et al., 2012, Brazil [56]
Thirst	0	20	3	20	Feguri et al., 2012, Brazil [56]
Acute atrial fibrillation	6	14	8	14	Feguri et al., 2019, Brazil [55]
Any post-surgery complication	nd	nd	2	14	Feguri et al., 2019, Brazil [55]
	8	28	12	29	Lee et al., 2017, Korea [59]
	34 ^†^	13	51	13	Tran et al., 2013, Canada [45]
Transfusion intra-operatively	7	14	7	14	Feguri et al., 2017, Brazil [54]
	6	28	4	29	Lee et al., 2017, Korea [59]
Transfusion post-operatively	6 *	14	5	14	Feguri et al., 2017, Brazil [54]
Pneumonia	3	14	3	14	Feguri et al., 2019, Brazil [55]
	0	13	0	13	Tran et al., 2013, Canada [45]
Reoperation	1	28	1	29	Lee et al., 2017, Korea [59]
Any complication intra-operatively	1	14	2	14	Feguri et al., 2017, Brazil [54]
EVA (encephalic vascular accident)	0	14	2	14	Feguri et al., 2017, Brazil [54]

* at ICU; ^†^ number of complications, nd—no data.

**Table 3:** Quantitative clinical outcomes that were not subjected to meta-analysis. Values are mean (standard deviation (SD)) or median (interquartile range (IQR)).

Outcomes	Treatment Group		Control Group		References/Country
	Mean (SD); Median (IQR)	*n*	Mean (SD); Median (IQR)	*n*	
Postoperative blood loss; mL	820 (670; 1010) ^^,†^	28	720(530; 830) ^^,†^	29	Lee et al., 2017, Korea [59]
Duration of mechanical ventilation; h	10 (9; 10) ^^^	20	10 (10; 15) ^^^	20	Sokolic et al., 2019a, Croatia [60]
	10 (10; 13.5) ^^^	20	9 (7.5; 10) ^^^	20	Sokolic et al., 2019b, Croatia [60]
	6 (4.1; 11.2) ^^^	13	6.7 (4.6; 12.5) ^^^	12	Tran et al., 2009, Canada [45]
Hospital length of stay; days	8.42 (7.79)	14	8.07 (4.5)	14	Feguri et al., 2017, Brazil [54]
	7.8 (1.4)	20	9.7(3.1)	20	Feguri et al., 2012, Brazil [56]
	11 (10; 14) ^^^	28	11 (0;14)	29	Lee et al., 2017, Korea [59]
	4 (4; 5) ^^^	12	5 (5; 6)	13	Tran et al., 2009, Canada [45]
ICU stay (hours)	48 (48; 72) ^^^	28	72 (48; 72) ^^^	29	Lee et al., 2017, Korea [59]
	24 (24; 48) ^^^	20	24 (24; 24) ^^^	20	Sokolic et al., 2019a, Croatia [60]
	24 (24; 24) ^^^	20	24 (24; 24) ^^^	20	Sokolic et al., 2019b, Croatia [60]
	21.3 (20.4; 22.9) ^^^	13	23.1 (21.8; 25.6) ^^^	12	Tran et al., 2009, Canada [45]
Duration of the surgery; min	220 (197.5; 242.5) ^^^	13	220 (195; 240) ^^^	13	Tran et al., 2009, Canada [45]
Postoperative blood transfusion; mL	200 (0; 295) ^^,†^	28	200 (0; 420) ^^,†^	29	Lee et al., 2017, Korea [59]
CPB duration; min	59 (50.5; 81.5) ^^^	13	61 (51.8; 72.5) ^^^	12	Tran et al., 2009, Canada [45]
Intra-operative gastric drainage; mL	26.8 (57.9)	50	16.3 (37.9)	51	Jarvela et al., 2008, Finland [57]
Post-operative gastric drainage; mL	88.8(75.4)	50	49.9 (63.4)	51	Jarvela et al., 2008, Finland [57]
Exogenous insulin intra-operatively; IU	5.9 (5.7)	20	7.5 (5)	20	Feguri et al., 2012, Brazil [56]
	0	28	0	29	Lee et al., 2017, Korea [59]
HOMA-IR endpoint	11.2 (8.2)	20	11.6 (7.6)	20	Feguri et al., 2012, Brazil [56]
	3.2 (0.9; 6.3) ^^^	12	1.8 (1.1; 3.8) ^^^	13	Tran et al., 2013, Canada [45]
	7.56 (1.48) ^‡^	9	7.2 (2.44)	9	Rapp-Kesek et al., 2007, Sweden [58]
CRP endpoint; mg.dL^−1^	3.75 (nd)	14	5.15 (nd)	14	Feguri et al., 2019, Brazil [55]
	−0.25 (-0.47; 0.21) ^^,§^	10	−0.1 (0.34; 0.39) ^^,§^	13	Tran et al., 2013, Canada [45]
IL-6 endpoint; pg·mL^−1^	1.92 (1.68–2.03) ^^,§^	7	1.79 (1.73; 1.91) ^^,§^	6	Tran et al., 2013, Canada [45]
Blood glucose post-operatively; mmol·L^−1^	8.15 (nd) ^¶,(a),(b)^	14	8.91 (nd)	14	Feguri et al., 2019, Brazil [55]
	6.44 (0.99) **^,(a)^	50	6.32 (nd)	51	Jarvela et al., 2008, Finland [57]
	7.8 (7.0; 8.2) ^^,††^	28	7.3 (7.8; 6.3) ^^^	29	Lee et al., 2017, Korea [59]
	6.7 (5.4; 7.1) ^‡‡^	13	6.6 (5.9; 8.1) ^^^	13	Tran et al., 2013, Canada [45]
	7.9 (0.5)^‡^	9	8.2 (0.7)	9	Rapp-Kesek et al., 2007, Sweden [58]

^^^ median (interquartile range); ^†^ postoperative 24 h; ^‡^ measured in the first POD (postoperative day); ^§^ as logarithm; CRP (mg·L^−1^), interleukin-6 (IL-6) (pg·mL^−1^); ^¶^ in the first hour in the postoperative period at the ICU; ** in the first six hours in postoperative period at the ICU; ^††^ median of six measurements over 48 h in the postoperative period; ^‡‡^ immediate postoperative period; ^(a)^ data from the graph; ^(b)^ converted from mg·dL^−1^ to mmol·L^−1^; CPB—cardiopulmonary bypass; CRP—C-reactive protein; HOMA-IR- Homeostasis Model Assessment for Insulin Resistance; nd—no data.

**Table 4:** GRADE (Grading of Recommendations, Assessment, Development and Evaluation) analysis: quality assessment of evidence.

Certainty Assessment							№ of patients		Effect		Certainty
№ of Studies	Study Design	Risk of Bias	Inconsistency	Indirectness	Imprecision	Other Considerations	OCH Loading (Treatment)	Fasting or Water (Control)	Relative (95% CI)	Absolute (95% CI)	
AC duration (min)											
3 (5 interventions)	randomized trials	not serious ^a^	not serious	not serious	not serious ^b^	publication bias strongly suspected ^c^	147	73	-	MD 6.388 lower (11.246 lower to 1.529 lower)	⨁⨁⨁◯ MODERATE
ICU stay (hours)											
4 (6 interventions)	randomized trials	not serious ^d^	serious ^e^	not serious	not serious ^f^	none	197	124	-	MD 25.925 SD lower (44.568 lower to 7.283 lower)	⨁⨁⨁◯ MODERATE
Exogenous insulin postoperatively (IU)											
3	randomized trials	not serious	not serious	not serious	serious ^g^	none	84	85	-	MD 4.523 lower (8.417 lower to 0.63 lower)	⨁⨁⨁◯ MODERATE
Inotropic drugs overall											
4 (6 interventions)	randomized trials	serious ^h^	not serious	serious ^i^	not serious	none	82/174 (47.1%)	51/101 (50.5%)	RR 0.795 (0.689 to 0.919)	104 fewer per 1000 (from 157 fewer to 41 fewer)	⨁⨁◯◯ LOW

CI: Confidence interval; MD: Mean difference; RR: Risk ratio; SMD: Standardized mean difference; AC—aortic clamping; ICU—Intensive Care Unit; ^a^ unclear risk of bias in selection bias, performance bias and detection bias in one study; ^b^ wide confidence intervals; ^c^ publication bias detected; ^d^ unclear risk of bias in selection bias, performance bias and detection bias in one study; ^e^ significant heterogeneity; ^f^ wide confidence intervals; ^g^ small sample size and wide confidence intervals; ^h^ unclear risk of bias in selection bias, performance bias and detection bias in two studies; ^i^ different time and oral dose of carbohydrate drink; ⨁⨁⨁⨁ high quality of the evidence; ⨁⨁⨁◯ moderate quality of the evidence; ⨁⨁◯◯ low quality of the evidence; ⨁◯◯◯ very low quality of the evidence.

## Supplementary Materials

https://www.mdpi.com/2072-6643/12/10/3105/s1, Table S1: The risk of bias assessment, Table S2: The results of continuous outcomes meta-analysis, Table S3: The results of categorical outcomes meta-analysis, Table S4. GRADE analysis: quality assessment of evidence.

## Abbreviations

AAF	Acute atrial fibrillation
AC	Aortic clamping
AMI	Acute myocardial infarction
CABG	Coronary Artery Bypass Grafting
CABG with CPB	Coronary Artery Bypass Grafting with Cardio-Pulmonary Bypass
CPB	Cardio-Pulmonary Bypass
CRP	C-reactive protein
DB	Double blinding
DM	Differences in means
ERAS	Enhanced Recovery After Surgery
GA with ETT	General Anaesthesia with Endotracheal Intubation
GI	Gastrointestinal
GLN	Glutamine
ICU	Intensive care unit
LOS	Length of stay
OC	Observed cases
OCH	Oral carbohydrate
OPCAB	Off-Pump Coronary Artery Bypass Grafting
POAF	Postoperative atrial fibrillation
PONV	Post-operative nausea and vomiting
PRISMA	Preferred Reporting Items for Systematic Reviews and Meta-Analyses
RCTs	Randomized controlled trials
ROB	Risk of bias
SB	Single blinding
SMD	Standardized mean difference
T2DM	Type 2 diabetes mellitus
VAS	Visual analog scale
