# Supplementary material for: The Effect of Preoperative Carbohydrate Loading on Clinical and Biochemical Outcomes after Cardiac Surgery: A Systematic Review and Meta-Analysis of Randomized Trials
Source: Nutrients. 2020 Oct 12;12(10):3105. doi: 10.3390/nu12103105 (PMC7600335; doi:10.3390/nu12103105)
Supplement: Supplementary file 1 [file nutrients-12-03105-s001.pdf]

Table S1. The risk of bias assessment.

Study	Random sequence generation (selection bias)	Allocation concealment (selection bias)	Blinding of participants and personnel (performance bias)	Blinding of outcome assessment (detection bias)	Incomplete outcome data addressed (attrition bias)	Selective reporting (reporting bias)	Other bias	Number of "Low" assessments	High quality (>3 low risk assessments) vs low (≤3 low risk assessments)
Feguri et al., 2017	L	L	L	U	L	L	L	6	**HQ**
Feguri et al., 2019	L	L	L	U	L	L	L	6	**HQ**
Feguri et al., 2012	L	U	L	U	L	L	L	5	**HQ**
Jarvela et al., 2008	L	H	H	U	L	L	L	4	**HQ**
Lee et al., 2017	L	L	L	U	L	L	L	6	**HQ**
Savluk et al., 2017<br>Savluk et al., 2017<br>Savluk et al., 2017	U	U	U	U	L	L	L	3	**LQ**
Sokolic et al., 2019a, Croatia<br>Sokolic et al., 2019b, Croatia	L	U	U	U	L	U	L	3	**LQ**
Tran et al., 2009	U	U	L	U	U	L	L	3	**LQ**
Rapp-Kesek et al., 2007	U	U	U	U	L	L	L	3	**LQ**

L - low risk of bias; U - unclear risk of bias; H - high risk of bias; HQ - high quality; LQ - low quality

Table S2. The results of continuous outcomes meta-analysis

Outcome/References	ENDPOINT DATA					
**Blood loss [ml]**						
Feguri et al., 2017, Brazil	**SMD (95% CI)**	**Z-value**	**Heterogeneity**	**Tau**	**Intercept (95%CI)†**	**Egger's test**
Savluk et al., 2017a, Turkey						
Savluk et al., 2017b, Turkey	0,239	1,912	Q=1,058	t^2=0	2,383 (-1,331; 6,098)	p=0,110
Savluk et al., 2017c, Turkey	(-0,006; 0,484)	p=0,056	p=0,787 (df=3)	t=0		
			I^2=0%			
**Duration of mechanical ventilation [hrs]**						
Feguri et al., 2017, Brazil	**SMD (95% CI)**	**Z-value**	**Heterogeneity**	**Tau**	**Intercept (95%CI)†**	**Egger's test**
Feguri et al., 2012, Brazil						
Savluk et al., 2017a, Turkey	-0,14	-1,204	Q=1,828	t^2=0	1,653 (-3,222; 6,529)	p=0,359
Savluk et al., 2017b, Turkey	(-0,368; 0,088)	p=0,229	p=0,767 (df=4)	t=0		
Savluk et al., 2017c, Turkey			I^2=0%			
**ICU stay [hrs]**						
Feguri et al., 2017, Brazil	**SMD (95% CI)**	**Z-value**	**Heterogeneity**	**Tau**	**Intercept (95%CI)†**	**Egger's test**
Feguri et al., 2012, Brazil						
Jarvela et al., 2008, Finland	-0,542	-4,307	Q=7,221	t^2=0,029	-2,163 (-8,695; 4,368)	p=0,409
Savluk et al., 2017a, Turkey	(-0,789; -0,295)	p<0,001	p=0,205 (df=5)	t=0,17		
Savluk et al., 2017b, Turkey			I^2=30,756%			
Savluk et al., 2017c, Turkey						
	**DM (95% CI)**	**Z-value**	**Heterogeneity**	**Tau**	**Intercept (95%CI)†**	**Egger's test**

	-25,925 (-44,568; -7,283)	-2,726 p=0,006	Q=28,802 p<0,001 (df=5) I^2=82,640%	t^2=333,603 t=18,265	-2,372 (-4,244; -0,500)	p=0,024*
**Duration of surgery [min]** Feguri et al., 2017, Brazil Feguri et al., 2012, Brazil	**SMD (95% CI)**	**Z-value**	**Heterogeneity**	**Tau**	**Intercept (95%CI)†**	**Egger's test**
Jarvela et al., 2008, Finland Lee et al., 2017, Korea Savluk et al., 2017a, Turkey Savluk et al., 2017b, Turkey Savluk et al., 2017c, Turkey	-0,033 (-0,331; 0,264)	-0,22 p=0,826	Q=14,781 p=0,022 (df=6) I^2=59,407%	t^2=0,093 t=0,3	2,223 (-6,296; 10,744)	p=0,531
**CPB duration [min]** Feguri et al., 2017, Brazil Feguri et al., 2012, Brazil	**SMD (95% CI)**	**Z-value**	**Heterogeneity**	**Tau**	**Intercept (95%CI)†**	**Egger's test**
Savluk et al., 2017a, Turkey Savluk et al., 2017b, Turkey Savluk et al., 2017c, Turkey	-0,019 (-0,247; 0,208)	-0,166 p=0,868	Q=2,273 p=0,686 (df=4) I^2=0%	t^2=0 t=0	3,233 (0,946; 5,521)	p=0,020**
**AC duration [min]** Feguri et al., 2017, Brazil Feguri et al., 2012, Brazil	**SMD (95% CI)**	**Z-value**	**Heterogeneity**	**Tau**	**Intercept (95%CI)†**	**Egger's test**
Savluk et al., 2017a, Turkey Savluk et al., 2017b, Turkey Savluk et al., 2017c, Turkey	-0,28 (-0,521; -0,038)	-2,266 p=0,023	Q=4,397 p=0,355 (df=4) I^2=9,021%	t^2=0,007 t=0,08	4,810 (2,525; 7,096)	p=0,006$
	**DM (95% CI)**	**Z-value**	**Heterogeneity**	**Tau**	**Intercept (95%CI)†**	**Egger's test**
	-6,388 (-11,246; -1,529)	-2,577 p=0,010	Q=3,988 p=0,408 (df=4) I^2=0%	t^2=0 t=0	3,821(3,008; 4,634)	p<0,001^

Exogenous insulin in the ICU/post-operatively [IU]	SMD (95% CI)	Z-value	Heterogeneity	Tau	Intercept (95%CI)†	Egger's test
Feguri et al., 2019, Brazil<br>Feguri et al., 2012, Brazil<br>Jarvela et al., 2008, Finland	-0,349<br>(-0,653; -0,044)	-2,246<br>p=0,025	Q=1,153<br>p=0,562 (df=2)<br>I^2=0%	t^2=0<br>t=0	-1,296 (-24,288; 21,695)	p=0,604
	**DM (95% CI)**	**Z-value**	**Heterogeneity**	**Tau**	**Intercept (95%CI)†**	**Egger's test**
	-4,523<br>(-8,417; -0,630)	-2,277<br>p=0,023	Q=1,264<br>p=0,532 (df=2)<br>I^2=0%	t^2=0<br>t=0	-1,445 (-26,582; 23,690)	p=0,598

† Egger’s regression intercept test for asymmetry of the funnel plots; AC – aortic clamping, CPB – cardio-pulmonary bypass, ICU – intensive care unit, ROB – risk of bias, T2DM -type 2 diabetes mellitus; * after adjustment (no values added to left of mean; two value adjusted to right of mean: DM=-16.7960, 95% CI: -32.962 to -0.629); ** after adjustment (no values added to left of mean and to right of mean); $ after adjustment (no values added to right of mean; one value added to left of mean: DM=-0.3364, 95% CI: -0.611 to -0.0613); ^ after adjustment (no values added to right of mean; one value added to left of mean: DM=-7.287, 95% CI: -12,9448 to -1.6294).

Table S3. The results of categorical outcomes meta-analysis.

Outcome/References	ENDPOINT DATA					
**AMI**	**RR (95% CI)**	**Z-value**	**Heterogeneity**	**Tau**	**Intercept (95%CI)†**	**Egger's test**
Feguri et al., 2017, Brazil<br>Lee et al., 2017, Korea<br>Tran et al., 2009, Canada	0,499<br>(0,086; 2,899)	-0,775<br>p=0,439	Q=2,002<br>p=0,367 (df=2)<br>I^2=0,111%	t^2=0,003<br>t=0,052	19,365 (-204,210; 242,941)	p=0,469
**Transfusion requirement**	**RR (95% CI)**	**Z-value**	**Heterogeneity**	**Tau**	**Intercept (95%CI)†**	**Egger's test**
Feguri et al., 2019, Brazil<br>Lee et al., 2017, Korea<br>Tran et al., 2009, Canada	0,808<br>(0,357; 1,827)	-0,513<br>p=0,608	Q=4,241<br>p=0,120 (df=2)<br>I^2=52,843	t^2=0,275<br>t=0,525	-1,054 (-76,702; 74,592)	p=0,888
**PONV**	**RR (95% CI)**	**Z-value**	**Heterogeneity**	**Tau**	**Intercept (95%CI)†**	**Egger's test**
Feguri et al., 2017, Brazil<br>Feguri et al., 2012, Brazil<br>Jarvela et al., 2008, Finland	0,993<br>(0,493; 2,0)	-0,018<br>p=0,985	Q=8,244<br>p=0,016 (df=2)<br>I^2=75,741%	t^2=0,263<br>t=0,513	-2,264 (-43,670; 39,140)	p=0,613
**Vasoactive drugs intra-operatively**	**RR (95% CI)**	**Z-value**	**Heterogeneity**	**Tau**	**Intercept (95%CI)†**	**Egger's test**
Feguri et al., 2017, Brazil	0,713	-1,067	Q=6,028	t^2=0,194	-4,117 (-33,466; 25,232)	p=0,325

Feguri et al., 2012, Brazil	(0,384; 1,327)	p=0,286	p= 0,049 (df=2)<br>I^2=66,821%	t=0,441		
Lee et al., 2017, Korea						
**Vasoactive drugs overall**	**RR (95% CI)**	**Z-value**	**Heterogeneity**	**Tau**	**Intercept (95%CI)†**	**Egger's test**
Jarvela et al., 2008, Finland	1,007	0,061	Q=7,654	t^2=0,033	-10,935 (-17,835; -4,034)	p=0,020
Savluk et al., 2017a, Turkey	(0,8; 1,268)	p=0,951	p=0,054 (df=3)	t=0,183		
Savluk et al., 2017b, Turkey			I^2=60,807%			
Savluk et al., 2017c, Turkey						
**Acute atrial fibrillation**	**RR (95% CI)**	**Z-value**	**Heterogeneity**	**Tau**	**Intercept (95%CI)†**	**Egger's test**
Feguri et al., 2017, Brazil	0,774	-0,817	Q=0,082	t^2=0	0,383 (-3,131; 3,898)	p=0,398
Feguri et al., 2012, Brazil	(0,418; 1,431)	p=0,414	p=0,960 (df=2)	t=0		
Tran et al., 2009, Canada			I^2=0%			
**Infectious complications**	**RR (95% CI)**	**Z-value**	**Heterogeneity**	**Tau**	**Intercept (95%CI)†**	**Egger's test**
Feguri et al., 2017, Brazil	0,591	-1,080	Q=0,282	t^2=0	-0,109(-2,242; 2,023)	p=0,845
Feguri et al., 2012, Brazil	(0,227; 1,536)	p=0,280	p=0,963 (df=3)	t=0		
Lee et al., 2017, Korea			I^2=0%			
Tran et al., 2009, Canada						
**Insulin treatment**	**RR (95% CI)**	**Z-value**	**Heterogeneity**	**Tau**	**Intercept (95%CI)†**	**Egger's test**
Feguri et al., 2012, Brazil	1,026	0,298	Q=4,685	t^2=0,009	0,311 (-4,606; 5,230)	p=0,810

Jarvela et al., 2008, Finland	(0,868; 1,212)	p=0,766	p=0,196 (df=3)	t=0,097		
Lee et al., 2017, Korea			I^2=35,968%			
Tran et al., 2009, Canada						
**Arrythmia**	**RR (95% CI)**	**Z-value**	**Heterogeneity**	**Tau**	**Intercept (95%CI)†**	**Egger's test**
Lee et al., 2017, Korea	0,897	-0,632	Q=2,930	t^2=0	-1,744(-7,700; 4,211)	p=0,334
Savluk et al., 2017a, Turkey	(0,641; 1,256)	p=0,528	p=0,403 (df=3)	t=0		
Savluk et al., 2017b, Turkey			I^2=0%			
Savluk et al., 2017c, Turkey						
**Inotropic drugs overall**	**RR (95% CI)**	**Z-value**	**Heterogeneity**	**Tau**	**Intercept (95%CI)†**	**Egger's test**
Feguri et al., 2012, Brazil	0,795	-3,116	Q=3,332	t^2=0	-0,822 (-2,289; 0,644)	p=0,194
Lee et al., 2017, Korea	(0,689; 0,919)	p=0,002	p=0,649 (df=5)	t=0		
Savluk et al., 2017a, Turkey			I^2=0%			
Savluk et al., 2017b, Turkey						
Savluk et al., 2017c, Turkey						
Tran et al., 2009, Canada						

† Egger's regression intercept test for asymmetry of the funnel plots; ROB – risk of bias, T2DM - type 2 diabetes mellitus; AMI - acute myocardial infarction; PONV - postoperative nausea and vomiting

Table S4. GRADE analysis: quality assessment of evidence

Certainty assessment							№ of patients		Effect		Certainty
№ of studies	Study design	Risk of bias	Inconsistency	Indirectness	Imprecision	Other considerations	OCH loading (treatment)	fasting or water (control)	Relative (95% CI)	Absolute (95% CI)	
**Blood loss (ml)**											
2 (4 interventions)	randomised trials	serious [a]	not serious	not serious	not serious	none	127	53	-	SMD **0.239 SD higher** (0.006 lower to 0.484 higher)	⊕⊕⊕◯ MODERATE
**Duration of mechanical ventilation (hrs)**											

Certainty assessment							№ of patients		Effect		Certainty
№ of studies	Study design	Risk of bias	Inconsistency	Indirectness	Imprecision	Other considerations	OCH loading (treatment)	fasting or water (control)	Relative (95% CI)	Absolute (95% CI)	
3 (5 interventions)	randomised trials	not serious [b]	not serious	not serious	not serious	none	147	73	-	SMD **0.14 SD lower** (0.368 lower to 0.088 higher)	⊕⊕⊕⊕ HIGH
**Duration of surgery (min)**											
5 (7 interventions)	randomised trials	not serious [c]	serious [d]	not serious	not serious	none	225	153	-	SMD **0.033 SD lower** (0.331 lower to 0.264 higher)	⊕⊕⊕◯ MODERATE
**CPB duration (min)**											
3 (5 interventions)	randomised trials	not serious [e]	not serious	not serious	not serious [f]	none	147	73	-	SMD **0.019 SD lower** (0.247 lower to 0.208 higher)	⊕⊕⊕⊕ HIGH
**Acute myocardial infarction**											

Certainty assessment							№ of patients		Effect		Certainty
№ of studies	Study design	Risk of bias	Inconsistency	Indirectness	Imprecision	Other considerations	OCH loading (treatment)	fasting or water (control)	Relative (95% CI)	Absolute (95% CI)	
3	randomised trials	not serious [g]	not serious [h]	serious [i]	serious [j]	none	1/55 (1.8%)	4/56 (7.1%)	**RR 0.499** (0.086 to 2.899)	**36 fewer per 1000** (from 65 fewer to 136 more)	⊕⊕◯◯ LOW
**Transfusion requirement**											
3	randomised trials	not serious [k]	serious [l]	serious [m]	serious [n]	none	16/55 (29.1%)	20/56 (35.7%)	**RR 0.808** (0.357 to 1.827)	**69 fewer per 1000** (from 230 fewer to 295 more)	⊕◯◯◯ VERY LOW
**PONV**											
3	randomised trials	not serious	serious [o]	not serious	serious [p]	none	49/84 (58.3%)	42/85 (49.4%)	**RR 0.993** (0.493 to 2.000)	**3 fewer per 1000** (from 251 fewer to 494 more)	⊕⊕◯◯ LOW
**Vasoactive drugs intra-operatively**											
3	randomised trials	not serious	serious [q]	not serious	serious [r]	none	29/62 (46.8%)	38/63 (60.3%)	**RR 0.713** (0.384 to 1.327)	**173 fewer per 1000** (from 372 fewer to 197 more)	⊕⊕◯◯ LOW

Certainty assessment							№ of patients		Effect		Certainty
№ of studies	Study design	Risk of bias	Inconsistency	Indirectness	Imprecision	Other considerations	OCH loading (treatment)	fasting or water (control)	Relative (95% CI)	Absolute (95% CI)	
**Vasoactive drugs overall**											
2 (4 interventions)	randomised trials	serious [s]	not serious [t]	not serious	not serious	none	111/163 (68.1%)	62/90 (68.9%)	**RR 1.007** (0.800 to 1.268)	**5 more per 1000** (from 138 fewer to 185 more)	⊕⊕⊕◯ MODERATE
**Acute atrial fibrillation**											
3	randomised trials	not serious [u]	not serious	serious [v]	serious [w]	none	11/47 (23.4%)	14/47 (29.8%)	**RR 0.774** (0.418 to 1.431)	**67 fewer per 1000** (from 173 fewer to 128 more)	⊕⊕◯◯ LOW
**Infectious complications**											
4	randomised trials	not serious [x]	not serious	serious [y]	serious [z]	none	5/75 (6.7%)	9/76 (11.8%)	**RR 0.591** (0.227 to 1.536)	**48 fewer per 1000** (from 92 fewer to 63 more)	⊕⊕◯◯ LOW
**Insulin treatment**											

Certainty assessment							№ of patients		Effect		Certainty
№ of studies	Study design	Risk of bias	Inconsistency	Indirectness	Imprecision	Other considerations	OCH loading (treatment)	fasting or water (control)	Relative (95% CI)	Absolute (95% CI)	
4	randomised trials	serious [aa]	serious [ab]	serious [ac]	not serious	none	74/111 (66.7%)	72/113 (63.7%)	**RR 1.026** (0.868 to 1.212)	**17 more per 1000** (from 84 fewer to 135 more)	⊕◯◯◯ VERY LOW
**Arrythmia**											
2 (4 interventions)	randomised trials	serious [ad]	not serious	not serious	not serious	none	41/141 (29.1%)	19/68 (27.9%)	**RR 0.897** (0.641 to 1.256)	**29 fewer per 1000** (from 100 fewer to 72 more)	⊕⊕⊕◯ MODERATE

CI: Confidence interval; **MD:** Mean difference; **RR:** Risk ratio; **SMD:** Standardised mean difference; **PONV**: postoperative nausea and vomiting; **CPB** – cardio-pulmonary bypass; a. unclear risk of bias in selection bias, performance bias and detection bias in one study; b. unclear risk of bias in selection bias, performance bias and detection bias in one study; c. unclear risk of bias in selection bias, performance bias and detection bias in one study; d. substantial I^2 ; e. unclear risk of bias in selection bias, performance bias and detection bias in one study; f. wide confidence intervals; g. unclear risk of bias in selection bias, detection bias and attrition bias in one study; h. moderate variance of point estimates across studies but confidence intervals (CI) overlap and very low I^2; i. different time and oral dose of carbohydrate drink; j. small sample size and wide confidence intervals; k. unclear risk of bias in selection bias, detection bias and attrition bias in one study; l. minimal overlap of confidence intervals (CI) and moderate I^2; m. different time and oral dose of carbohydrate drink; n. small sample size and wide confidence intervals; o. high value of I^2 ($p<0,05$); p. small sample size and wide confidence intervals; q. minimal overlap of confidence intervals (CI) and substantial value of I^2 ($p<0,05$); r. small sample size and wide confidence intervals; s. unclear or high risk of bias in selection bias, performance bias and detection bias in two studies; t. overlap of confidence intervals (CI) and moderate value of I^2 ($p=0,054$); u. unclear risk of bias in selection bias, detection bias and attrition bias in one study; v. different time and oral dose of carbohydrate drink; w. small sample size and wide confidence intervals; x. unclear risk of bias in selection bias, detection bias and attrition bias in one study; y. different time and oral dose of carbohydrate drink; z. small sample size and wide confidence intervals; aa. unclear or high risk of bias in selection, performance, detection or attrition bias in two studies; ab. minimal overlap of confidence intervals (CI); ac. different time and oral dose of carbohydrate drink; ad. only one high-quality (HQ) study;
